# REDCap as an accreditation tool for academic programs in clinical research: A case study

**DOI:** 10.1017/cts.2024.615

**Published:** 2024-10-31

**Authors:** Barbara Tafuto, Doreen W. Lechner

**Affiliations:** 1 Department of Health Informatics, Rutgers School of Health Professions, New Brunswick, NJ, USA; 2 New Jersey Alliance for Clinical and Translational Science, New Brunswick, NJ, USA

**Keywords:** Accreditation, clinical research management, CTSA, education, REDcap

## Abstract

The Master of Science in Clinical Research Management program at Rutgers Biomedical Health Sciences underwent significant restructuring aligned with the Clinical and Translational Science Award funding parameters. This evolution necessitated formal evaluation through accreditation by the Commission on Accreditation of Allied Health Education Programs. The years-long accreditation process posed challenges, particularly regarding the collection of course learning outcomes data aligned with accreditation competency standards. The objective of this special communication is to report the rationale behind pursuing accreditation for clinical research degrees, the data collection challenges during the accreditation process, and a potential solution. In order to address existing university metric data gaps, Research Electronic Data Capture (REDCap) software was used to develop a data collection tool that streamlined the accreditation process and reduced the administrative burden. REDCap was effective in allowing faculty to self-report 3 years of course outcomes data for accreditation. There was an elevated level of user satisfaction compared to alternative data collection methods. A SWOT analysis identified the strengths and weaknesses of using REDCap, emphasizing strengths in functionality that include customizability, data validation, and compliance with regulatory standards. Overall, the advantages of leveraging REDCap for accreditation data collection, including customization, data security, and user-friendliness outweigh the key disadvantage of REDCap, which is its limited reporting capabilities.

## Introduction

Academic program accreditation benefits all aspects of higher education because the process requires continuous data-driven assessments designed to document and improve the rigor of a program’s curriculum and its level of student success [1]. While licensed careers traditionally require an accreditation component within the degree, there also exist programs where accreditation is not mandated but is pursued to ensure the professionals graduating from such programs meet a recommended set of competencies. This paper outlines a rationale for pursuing accreditation for a clinical research management master’s program and describes and assesses the value of customizing a supplementary data collection tool for reducing the accreditation process’s administrative burden.

### A rationale for clinical research professional accreditation

In 2019, Rutgers Biomedical Health Sciences (RBHS) along with Princeton University and New Jersey Institute of Technology were awarded the Clinical and Translational Science Award (CTSA) and formed the New Jersey Alliance for Clinical and Translational Science (NJ ACTS). Once funded, NJ ACTS recommended restructuring educational programs relating to clinical and translational science (CTS) to support the training and education goals aligned with the CTSA funding parameters [2]. To that end, one of the primary goals for the workforce development core for the initial NJ ACTS grant (2019–2024) was to ensure that (a) degree programs in CTS were available; (b) these programs included clear, universally accepted professional core competencies; and (c) evaluation for these core competencies were well defined [3].

As a result of this CTSA alignment, the RBHS Master of Science in Clinical Trial Sciences evolved into the Master of Science in Clinical Research Management (MS CRM). The evolution of the MS CRM program included a consolidation of four industry-diverse specialty track options to three more condensed CRM-related track options: (a) Clinical Research Management, (b) Drug Safety/Pharmacovigilance, and (c) Academic Clinical Research Management. Courses were restructured between core courses and electives to ensure that all clinical research professional competencies were met within each track’s core curriculum, while the electives allowed students to explore more diverse special topics across clinical research. While the MS CRM aligns with the development of competent holistic clinical research professionals, this realignment was developed with the added goal of training a “world-leading, globally connected workforce” equipped with “the skills needed to excel in translational science,” as outlined by the CTSA and National Institute of Health [3]. These adjustments, along with the driving force of NJ ACTS and CTSA goals, formed the need to formally evaluate the level and quality of the newly developed MS CRM curriculum through the respective accrediting organization.

### CAAHEP accreditation process

The Commission on Accreditation of Allied Health Education Programs (CAAHEP) was established in 1994 through the support of the American Medical Association as an independent agency to assume accrediting responsibilities for Allied Health Professions [4,5]. CAAHEP has 25 committees on accreditation, including the Committee on Accreditation of Academic Programs in Clinical Research (CAAPCR). The RBHS MS CRM program set their accreditation goals to meet the CAAPCR accreditation guidelines [6]. The CAAHEP process begins with a request for accreditation services by the program, the submission of a comprehensive self-study report, a review and site visit of the self-study report by CAAPCR, followed by the program’s response to the site visit findings. Once the program’s response is received, CAAPCR reviews the program’s compliance with the standards and accreditation recommendation and in turn submits their recommendation to CAAHEP who provides the program’s accreditation status. After accreditation occurs CAAHEP is responsible to continually monitor the program’s compliance with published criteria. Presently, three MS clinical research-based programs are accredited by CAAHEP, one of which has CTSA funding [7]. While MS clinical research professional-based program accreditation is limited among US institutions of higher education, NIH CTSA funding guidelines underscore that high standards and rigorous program evaluation are critical to robust clinical and translational workforce development [3]. In 2020, the RBHS MS CRM program began identifying, collecting, and organizing needed program data in preparation for the application process for accreditation through CAAHEP.

One aspect of preparing for accreditation begins at the most basic level with individual course assessments performed by faculty. These course assessments require comprehensive data supporting the alignment of the course objectives and student learning outcomes to the assessments with program-level and career-level outcomes. Typical data that are collected and evaluated for alignment are noted in Table 1: Course learning outcome form data fields. This table depicts the fields on an in-house course learning outcome form and the requisite field options for each data field.

**Table 1.** Course learning outcome form data fields

Data Field	Description Data Field Options
Course objectives	One course may have between 6 and 20 objectives that need to be aligned with all assignments
Course measures	All measures which fulfill respective course objectives (assignment, test, etc. – with brief description)
Is this measure a direct (faculty-centered direction) or indirect (student-centered inquiry) measure?	2 Options: Direct or indirect
At what level is this objective taught	3 Options: Introduced, reinforced, or mastered
Which School Learning Outcome is this course objective aligned with?	29 Outcomes among 10 categories (more than 1 may be selected)
Which Program-Level Outcomes is this course objective aligned with?	25 Outcomes among three categories (more than 1 may be selected)
Which Joint Taskforce Competency Domain is this course objective aligned with?	8 Domains (more than one may be selected)
Which Joint Taskforce Competency is this course objective aligned with?	Within the 8 domains, there are 49 competencies (more than 1 may be selected)
Did student meet performance standard (80% of students attained 80%)	Calculated based on the number of Master of Science in Clinical Research Management students achieving at least 80% on each measure
If success performance is not met, provide corrective action	Narrative collected

Along with the data sources collected for each course, CAAPCR requires 3 years of annual program assessment reports to be included in the self-study application packet. Graduate-level degree programs can have between 13 and 30 courses offered to its students that would require assessment. Full-time faculty can teach between six and eight courses each year. The methods and processes for faculty to identify, collect, and organize disparate course data based on these requirements can be an overwhelming responsibility amid their existing course workload, community service, and scholarly activities. Depending on the number of courses and assessments in a single course, the measurement of success can involve the evaluation of hundreds of data points for each faculty member to consider. The burden the accreditation process places on a program to track multiple years of course assessments can include challenges relating to data completeness, data accuracy, and data analysis of each course assessment. Ongoing similar cyclical assessments are required to maintain the CAAHEP’s accreditation status, so data also need to be safely stored and continually expanded.

One advantage to the CAAHEP Accreditation process for the MS CRM Program at RBHS was that the university’s commercial system used for collecting program metrics was already collecting a portion of the required data. The major challenge arose when it was understood the university system did not have viable provisions for the collection of needed data regarding clinical research professional career-level outcomes predicated on Standards and Guidelines for the Accreditation of Educational Programs in Clinical Research Curriculum for Educational Programs in Clinical Research. The competencies represent a critical factor related to the data collected for CAAHEP accreditation. These critical data points for accreditation include eight domains: (1) Scientific Concepts and Research Design, (2) Ethical and Participant Safety Considerations, (3) Medicines Development and Regulation, (4) Clinical Trial Operations (Good Clinical Practice), (5) Study and Site Management, (6) Data Management and Informatics, (7) Leadership and Professionalism, and (8) Communications and Teamwork. Each of these domains has among them a total of 50 competencies where CAAHEP accreditation requires course assignments to identify alignment to the specific competencies [8–10]. This can lead to more than 116 different datapoints for each assignment in each course.

### RBHS MS CRM: A case study in barriers to career outcomes data collection

In Spring 2021, a NJ ACTS workforce development internship project was undertaken with the objective of mapping and aligning the Joint Taskforce for Clinical Trial Competency Framework (JTF) to assignments in 31 courses in the RBHS MS CRM program [8]. The internship was an attempt to prepare for the more formal process of accreditation and understand potential barriers of gaps and data collection in general. Over a 180-hour internship period, the project resulted in JTF alignment and data capture for three of the 31 MS CRM courses originally planned. An assessment of barriers to this mapping activity identified: (a) the utilization of an Excel spreadsheet as the data capture tool slowed down the data collection of the mapping process considerably and (b) challenges the faculty had finding the time to meet the milestones required for this data collection process. Based on the limited 2021 NJ ACTS Workforce Development internship outcomes, the prospect of JTF competency data collection and organization for CAAHEP accreditation presented a considerable obstacle. Understanding the obstacles presented from the internship and that the accreditation process in general has been reported to induce anxiety, sleepless nights, and long days for all involved, a team within the RBHS MS CRM program collaborated to alleviate the anticipated stress of this burden [11].

After a collaborative discussion within the program, it was determined that a supplemental accreditation data collection tool using Research Electronic Data Capture (REDCap) software would be designed and developed [12]. The objectives of this data collection tool development were to (a) simplify the outcomes alignment burden on faculty and (b) increase the efficiency in which the data is collected [12]. The following Methods and Results sections outline (a) the RBHS MS CRM experience developing the tool using REDCap, (b) the understanding of user preferences in the data collection process, and (c) a description of the generalized data dictionary.

## Methods

For the purposes of this project, the REDCap software hosted at Rutgers New Jersey Medical School was configured to collect customized RBHS MS CRM course data through simple prompts and descriptive instructions that would allow end users to enter data through a more seamless workflow while also allowing administrators to review progress and results in a more structured and real-time process.

The project build began with a review of credentialing requirements and CAAPCR standards and guidelines to identify the specific data gaps not captured by the university-wide data collection system. It was determined that the career outcomes data, identified as clinical research competencies, were the foundation of the supplemental REDCap build.

Once data needs were identified, the goal for the data collection tool build was to create a user-centric design allowing for a streamlined process. A concerted effort was made to reduce the free text responses with piping functions, limit unnecessary questions using branching logic, and improve efficiency using repeatable forms.

Once data collection for the 3 years was concluded, seven front-end users completed a brief survey on their data entry experiences, and two key backend users conducted a SWOT analysis of the overall functionality of the tool for accreditation purposes.

## Results

These results describe (a) key functionalities of the tool, (b) the data collection experience including a summary of the user survey, and (c) a SWOT analysis of the process.

### Key functionalities of the supplemental accreditation data collection tool

The data collection tool was completed using two instruments, one foundational instrument called, “Course Identifiers,” and the other repeatable instrument called, “Course Accreditation Objective Assignments.” The instrument as a whole was developed to allow the template to be expanded to other learning outcomes within the same program or translated to other programs.

The “Course Identifier” instrument collected standard course data and included 48 fields that collected common course data points and had an auto continue function to bring the user to the Course Accreditation Objective Assignments. These data points included two types of data, (a) data that identified the course such as course name, course code, semester, year, and instructor; and (b) data that needed to be recalled through the system to simplify upcoming more detailed data collection activities such as course objectives and student enrollment numbers.

The “Course Accreditation Objective Assignments” instrument collected the information for the various outcomes and student performance. This instrument’s repeatable function allowed users to enter data for one assignment after another in a course without having to reenter the course identifiers. This also allowed for unlimited assignment data entries. This instrument could only be accessed once the foundational data had been entered in the “Course Identifier” instrument. Since data captured in the “Course Identifier” instrument needed to migrate to the “Course Accreditation Objective Assignments” instrument piping logic was used. Piping logic is a programmable function within REDCap that identifies and inserts previously collected data into text on another data collection form, which improves precision and efficiency [13]. Migrating course objectives made it possible for users to have a list of the objectives identified for the course auto populated into the Course Accreditation Objective Assignments instrument, making it possible to select the objective that aligned with the assessment rather than having to type it in as a text entry.

### Data collection experience

Over the years of preparing for this process, faculty, administrators, and interns participated in data collection activities using different formats for their course learning outcomes. The process was always conducted using standardized course learning outcome templates. These templates encompassed essential information as exhibited in Table 1.

Data Collection included 87 courses over 3 years, with a range of 3–20 objectives that classified more than 1300 assignments among 8 clinical research professional domains, 50 competencies, and the 3 levels of learning. Student outcomes data from course gradebooks were also calculated and recorded. The time to complete for the data collection process using the REDCap tool versus other formats per course was more efficient. User satisfaction for the REDCap tool garnered an average score of 7.5 using a 1 through 10 Likert scale. All users preferred using REDCap over previously used Word Document and Excel formats. User comments supporting the REDCap process focused on improved time to complete and ease of process. User recommendations focused on additional autofill technology and navigation challenges.

### SWOT analysis

The REDCap tool architect and the data analyst of this project reviewed the methods and processes involved in the tool development and backend functionality to better understand the strengths, weaknesses, opportunities, and threats to the application of REDCap data capture for supplemental data accreditation purposes.

The strengths of this project began with the fundamental user interface for the collection tool build which required a manageable level of expertise by the accreditation team members and no need for external technology resources. Modifications in the data collection tool or reports could be handled within the internal accreditation team which streamlined the project build. The customizability of REDCap allowed for the development of a data collection tool that was able to meet the CAAHEP accreditation data needs that were not addressed in the university system. It also allows for data collection tool adjustments in the event data needs change. Built-in data validation features such as auto-calculation, drop-down options, smart variables, piping, and branching logic helped reduce errors. The data security and Health Insurance Portability and Accountability Act (HIPAA) compliance provided by REDCap also ensures that potential university proprietary data is protected from unauthorized access or security breaches.

The most notable weakness in this project build is that the limited REDCap reporting functionality required the accreditation team to export data files and rebuild the reports for a more structured analytical presentation. This limitation handicapped the final report preparations. Another weakness was that faculty cannot carry over standard data from the previous year. While the initial build was designed so faculty would need to reenter the standard data for each course in order to encourage review and evaluation of the course each semester, faculty have noted that the workload for this task would be significantly reduced if they could autofill the standard data from semester to semester.

The fact that REDCap is a free data capture system available to institutions of higher education is an opportunity. It allows for the framework of this tool to be shared and utilized by academic programs at other universities looking to submit their CAAHEP accreditation. The framework also establishes a methodology for modifications for other types of accreditations. The fact that the system is housed within the academic program allows faculty to query the existing data in an ongoing basis to learn more about competency representation and detailed student performance on assignments.

One of the greater threats to the use of REDCap is that the university could decide to no longer support this software, wherein the ability to work within this tool would be jeopardized. While there is only a need for limited expertise in REDCap build, the loss of personnel familiar with REDCap can also impact the continued or seamless use of the tool in an ongoing basis.

Table 2 summarizes the key points and provides a clear overview of the strengths, weaknesses, opportunities, and threats related to the REDCap tool and its application for supplemental data accreditation.

**Table 2.** SWOT analysis

Strengths	Weaknesses
**User Interface:** Manageable expertise, reduce need to external tech resources. I**nternal Handling**: Modifications and report adjustment managed internally by the program team, streamlining project build. **Customizability:** Adaptable to meet specific Commission on Accreditation of Allied Health Education Programs (CAAHEP) accreditation data needs, with flexibility for future changes. **Built-in Data Validation**: Features like auto-calculation, drop-down options, smart variables, piping, and branching logic reduce error. **Data Security and Health Insurance Portability and Accountability Act (HIPPA) Compliance:** Ensures protection of proprietary data from unauthorized access or breaches.	**Limited Reporting Functionality**: Requires exporting data files and rebuilding reports, which hampers structured analytical presentation. **No Autofill Functionality:** Faculty cannot carry over standard data for a course from the previous semester.
Opportunities	Threats
**Free System**: Research Electronic Data Capture (REDCap) is available at no cost to higher education institutions, facilitating its use for other academic programs seeking accreditation. **Framework Sharing**: Provides a basis for other types of accreditation methodologies and allows for ongoing faculty queries on data to assess competency and performance.	**University Support Risk**: University discontinuing support for software, potentially jeopardizing continued use of the tool. **Loss of Expertise:** Departure of personnel skilled in software could impact ongoing or seamless use of the tool.

## Discussion

REDCap is a secure, web-based software platform designed to support data capture for research studies, providing (1) an intuitive interface for validated data capture, (2) audit trails for tracking data manipulation and export procedures, (3) automated export procedures for seamless data downloads to common statistical packages, and (4) procedures for data integration and interoperability with external sources” [14]. REDCap is a CTSA-funded data collection software application shared by Vanderbilt University and adopted by over 6,000 partners among 150+ different countries [15]. Due to its compliance with 21 Code of Federal Regulations Part 11, Federal Information Security Modernization Act, HIPPA, and General Data Protection Regulation for the Regulation (EU) 2016/679, REDCap finds its utility in a wide range of CTS domains including but not limited to fundamental scientific research, gathering data for clinical trials, registries, cohort studies, conducting quality assessments for clinical practices, facilitating comparative effectiveness trials, administering patient questionnaires, empowering clinical decision support systems, and providing operational or administrative assistance [13,14,16–21].

By utilizing standardized school-developed course learning outcome templates, the data were easily translated into the REDCap user interface forms. Due to the layout of REDCap, data entry became more efficient with gaining familiarity with terminology and the clinical research professional competencies. It also eliminated the need to refer to the various outcome reference measurement tools since they were all embedded into REDCap.

Standardizing the data to each objective which was linked to a number of assessments per course, allowed for a seamless analysis. Although REDCap report functionality was a challenge since the report format did not align with the report templates recommended in the Self Study Report Format, the customized reports within REDCap provided a distinct view of each individual course, along with a variety of other views of the data. This enabled opportunities for direct comparisons among course performance along with in-depth evaluations of individual clinical research professional competencies and potential redundancies. Student success rates allowed for gap analysis as well as evaluate process improvement opportunities across course offerings. When selecting a suitable data collection format for this project, efficiency in data entry and data analysis was a crucial consideration. Analysis of the end-user survey data and the SWOT analysis support the use of REDCap over other formats. Leveraging REDCap as a CAAHEP accreditation data collection tool offers numerous benefits in terms of customization, data validation, security, and an acceptable level of user-friendliness. Institutions looking to model the process outlined in this communication should consider the needed personnel for development and limited reporting capabilities. Future applications relating to this type of administrative use of REDCap could be expanded to other academic program assessment needs.
